# Editorial: Applications of RNA-seq in cancer and tumor research

**DOI:** 10.3389/fgene.2023.1331576

**Published:** 2023-11-14

**Authors:** Jidong Lang, William C. Cho, Tao Huang, Taoyang Wu, Junlin Xu

**Affiliations:** ^1^ Department of Bioinformatics, Qitan Technology (Beijing) Co., Ltd., Beijing, China; ^2^ Department of Clinical Oncology, Queen Elizabeth Hospital, Hong Kong SAR, China; ^3^ Shanghai Institute of Nutrition and Health, Chinese Academy of Sciences (CAS), Shanghai, China; ^4^ School of Computing Science, University of East Anglia, Norwich, United Kingdom; ^5^ College of Computer Science and Electronic Engineering, Hunan University, Changsha, China

**Keywords:** RNA sequencing, cancer research, next-generation sequencing, biomarker, machine learning

Over the past decade, RNA sequencing, commonly referred to as RNA-Seq, has emerged as a powerful and significant tool, leading to remarkable advancements in the fields of cancer and tumor research. Presently, RNA-Seq is extensively employed in molecular biology, playing a pivotal role in enhancing our comprehension of genome functions, particularly those relevant to cancer research (Stark et al., 2019). Notably, it has become an indispensable tool for conducting whole-transcriptome analysis, enabling the study of differential gene expression and differential splicing of mRNAs. Transcript isoform expression and usage, a key source of variation between healthy and cancerous or diseased tissues in various medical conditions, can be effectively investigated using this technique (Gonzalez and McGraw, 2009). Undoubtedly, the advent of sequencing technologies, such as next-generation sequencing (NGS) and nanopore sequencing, has facilitated comprehensive transcriptome analysis, leading to significant breakthroughs in cancer and tumor research. These technologies have enabled the examination of single-cell gene expression, translation, RNA structure, and spatial transcriptomics. Nanopore sequencing stands out for its ability to deliver full-length transcripts accurately and to identify and quantify multiple isoforms, making it particularly valuable for cancer research. This technology has been successfully applied in the study of various cancer types, including leukaemia, breast cancer, ovarian cancer, and lung cancer (de Jong et al., 2017; Minervini et al., 2017; Suzuki et al., 2017). Some studies have even suggested that RNA-Seq has the potential to revolutionize the analysis of eukaryotic transcriptomes (Wang et al., 2009). Its ability to investigate diverse aspects of RNA biology in cancer and tumors is critical for developing a functional understanding of the genome, studying development processes, and identifying molecular dysregulation underlying cancer and other diseases. Consequently, RNA-Seq has already assumed a vital role in practical clinical applications (Byron et al., 2016; Haque et al., 2017). In this Research Topic, we have compiled 11 papers that highlight several frontiers in the role of RNA-Seq in cancer and tumor research.

Du et al. focused on investigating the genomic effects of high-dose single-shot radiotherapy with the aim of providing a dynamic map for non-small cell lung cancer (NSCLC). The authors employed whole-transcriptome sequencing to elucidate molecular-level changes in A549 and H1299 cell lines exposed to 10 Gy X-rays at different time points, comparing them to a no radiation group, and found dynamic changes following radiation therapy within 48 h. Their findings emphasized key molecules and pathways involved in NSCLC after high-dose single-shot radiotherapy. This study contributes to enriching the content of radiobiology in precision radiation oncology.

Jin et al. utilized a published single-cell transcriptomics profile to deconvolute the abundance of cell types among paired plasma samples from colorectal cancer patients who underwent tumor-ablative surgery. Their objective was to identify the tissue-specific contributions of circulating cell-free RNA (cfRNA) transcriptomic profiles. Furthermore, they validated differentially expressed cfRNAs using RNA-Seq. The authors observed a significant decrease in the transcriptomic component from intestinal secretory cells in post-surgical cfRNA samples. They also found consistent expression of *HPGD*, *PACS1*, and *TDP2* between cfRNA and tissue samples, indicating the potential of these markers for minimal residual disease (MRD) testing, which involves profiling remnants cancer cells after or during treatment.

Song et al. identified key genes associated with cuproptosis and ferroptosis (*POR*, *SLC7A5* and *STAT3*) involved in sepsis-induced cardiomyopathy (SIC). Additionally, they explored therapeutic drug candidates. This work holds promise for the development of treatments for SIC.

Nousiainen et al. conducted RNA-Seq analysis on xenografts and immortalized cell lines to gain insights into the pathobiology of hepatoblastoma (HB). Through protein-protein interaction analysis, they identified ubiquitination as a key dysregulated pathway in HB. The study also revealed the potential prognostic utility of *UBE2C* in HB and highlighted the ubiquitin pathway as a potential therapeutic target of the disease.

Zhu et al. provided a comprehensive summary of the main methods for detecting circulating tumor DNA (ctDNA), including PCR-based sequencing and NGS, along with their respective advantages and disadvantages. Additionally, the authors reviewed the significance of ctDNA analysis in guiding adjuvant therapy and predicting relapse in lung, breast, and colon cancers, among others. Despite the existing challenges in MRD detection, the feasibility of ctDNA as a detection method and the revolutionary potential of ctDNA-based liquid biopsies offer a promising approach to cancer monitoring.

Xie et al. developed a prognostic risk model and identified immune ferroptosis-related genes with independent prognostic value using procedural algorithm analysis. Their findings demonstrated significant correlations between immune scores, immune checkpoints, and chemotherapeutic agents with prognostic models. These features were subsequently considered as independent prognostic factors for predicting overall survival (OS) and clinical treatment response in breast cancer patients. This study provides a better understanding of the contribution of immune ferroptosis-related genes in breast cancer and highlights their potential as prognostic markers and therapeutic targets.

Wang et al. employed consensus clustering to identify two disulfidptosis-molecular subtypes in breast cancer, with differing OS outcomes. Subsequently, the authors developed a prognostic signature based on differentially expressed genes related to disulfidptosis, which demonstrated improved predictive capabilities for patient survival and provided preliminary insights into the relationship between the risk model and the immune landscape. This study offers valuable prognostic predictions for breast cancer patients, with prognostic signatures closely associated with the tumor microenvironment, potentially informing clinical treatment decisions.

Niu et al. proposed a microRNA (miRNA) and small molecule association prediction model, named GCNNMMA, by integrating graph neural networks and convolutional neural networks. This model inspired by ensemble learning, demonstrated superior cross-validation results compared to other comparative models, suggesting the effectiveness of GCNNMMA in mining the relationship between small molecule drugs and disease-relevant miRNAs. GCNNMMA holds promise as a valuable tool for exploring the associations between small molecules and miRNAs in disease contexts.

Li et al. developed a novel ensemble model, called autoencoder-assisted graph convolutional neural network (AE-GCN), that combined autoencoder and graph convolutional neural network techniques to identify accurate and fine-grained spatial domains. In cancer datasets, AE-GCN successfully identified disease-related spatial domains, revealing more heterogeneity than traditional histological annotations. Moreover, AE-GCN facilitated the discovery of novel differentially expressed genes with significant prognostic relevance. This study demonstrates the ability of AE-GCN to unveil complex spatial patterns from spatially resolved transcriptomics data.

Chen et al. addressed the lack of a specialized database focusing on alternative splicing events (ASEs) in esophageal squamous cell carcinoma (ESCC) and the underrepresentation of long non-coding RNAs (lncRNAs) in ESCC molecular mechanisms with the development of a database, called DASES. DASES provides comprehensive insights into ASEs in ESCC, encompassing both lncRNAs and mRNAs, thereby enhancing the understanding of ESCC molecular mechanisms and serving as a valuable resource for the ESCC research community.

Su et al. introduced a machine learning-based method, called LDAenDL, which utilizes an ensemble of deep neural networks and LightGBM, to detect potential lncRNA biomarkers for lung cancer and neuroblastoma. The authors demonstrated that LDAenDL outperformed classical LDA prediction methods, and identified new potential biomarkers for these diseases. The application of LDAenDL may facilitate the development of targeted therapies for lung cancer and neuroblastoma.

In summary, these papers demonstrate the diverse applications of RNA-Seq in cancer and tumor research. The studies utilize RNA-Seq to identify differentially expressed genes, explore molecular mechanisms, and identify potential therapeutic targets in various types of cancer. The findings contribute to our understanding of cancer biology and highlight the potential of RNA-Seq in improving cancer diagnosis, prognosis, and treatment.
